# The Individual Blood Cell Telomere Attrition Rate Is Telomere Length Dependent

**DOI:** 10.1371/journal.pgen.1000375

**Published:** 2009-02-13

**Authors:** Katarina Nordfjäll, Ulrika Svenson, Karl-Fredrik Norrback, Rolf Adolfsson, Per Lenner, Göran Roos

**Affiliations:** 1 Department of Medical Biosciences, Pathology, Umeå University, Umeå, Sweden; 2 Department of Clinical Sciences, Psychiatry, Umeå University, Umeå, Sweden; 3 Psychiatric Clinic, Sunderby Hospital, Luleå, Sweden; 4 Department of Radiation Sciences, Oncology, Umeå University, Umeå, Sweden; Medical Research Council Human Genetics Unit, United Kingdom

## Abstract

Age-associated telomere shortening is a well documented feature of peripheral blood cells in human population studies, but it is not known to what extent these data can be transferred to the individual level. Telomere length (TL) in two blood samples taken at ∼10 years interval from 959 individuals was investigated using real-time PCR. TL was also measured in 13 families from a multigenerational cohort. As expected, we found an age-related decline in TL over time (r = –0.164, *P*<0.001, n = 959). However, approximately one-third of the individuals exhibited a stable or increased TL over a decade. The individual telomere attrition rate was inversely correlated with initial TL at a highly significant level (r = –0.752, *P*<0.001), indicating that the attrition rate was most pronounced in individuals with long telomeres at baseline. In accordance, the age-associated telomere attrition rate was more prominent in families with members displaying longer telomeres at a young age (r = –0.691, *P*<0.001). Abnormal blood TL has been reported at diagnosis of various malignancies, but in the present study there was no association between individual telomere attrition rate or prediagnostic TL and later tumor development. The collected data strongly suggest a TL maintenance mechanism acting *in vivo*, providing protection of short telomeres as previously demonstrated *in vitro.* Our findings might challenge the hypothesis that individual TL can predict possible life span or later tumor development.

## Author Summary

An age-dependent telomere shortening has been frequently observed in cross-sectional studies on human blood cells. Telomerase is an enzyme capable of lengthening telomeres, and it is activated in most tumor cells in order for them to become immortalized. This is one of the first longitudinal studies on telomere length, investigated in human blood samples taken at two occasions with approximately 10 years between them. An interesting finding was that the individual telomere length in the first blood sample was highly correlated with telomere attrition rate. Thus, individuals displaying the longest telomeres at baseline showed the most rapid telomere shortening over time and vice versa. This was also observed at the family level when exploring a multigenerational cohort. These results are in concordance with the fact that telomerase seems to preferentially act on the shortest telomeres in cultivated cells and provide fundamental knowledge for general telomere and cell biology. Because one part of the cohort developed tumors after the second blood draw, we had the opportunity to examine whether telomere attrition rate differed in tumor patients compared with controls, but no such indication was observed. However, for prostate cancer, short telomere length ≥9 years before diagnosis seemed to predict death.

## Introduction

Telomeres are protective end structures of the chromosomes. Telomere length is dictated partly by hereditary [1]–[6] and partly by environmental [7],[8] and epigenetic factors [9]. The hereditary impact on TL has been estimated to range between 36–84% [3]–[6]. An equally strong telomere length inheritance was reported for monozygotic (MZ) as for dizygotic (DZ) twin pairs, indicating that the correlation in TL was mainly due to shared environmental factors [10]. In contrast, relatively minor environmental effects on TL during life were suggested in MZ twins where identical homologue telomeres differed less in TL compared to the two alleles within one individual [1],[2]. Regarding the influence of life style and environment on telomere maintenance, the published data are conflicting and no consensus has been reached concerning the impact of e.g. smoking, blood pressure or serum lipids on TL (literature overview in [11]).

Patients with smoking associated malignancies, such as human bladder, head and neck, lung, and renal cell cancers, have been shown to display shorter blood TL at diagnosis compared to controls [12],[13]. Short blood TL has therefore been suggested as a predisposition factor for these cancer types. For breast cancer, no difference in blood TL between patients and controls was found in one study [14], whereas we recently reported longer telomeres in peripheral blood cells of breast cancer patients and, furthermore, that long blood TL indicated a poor survival [15]. Numerous studies have shown an inverse correlation between blood cell TL and age [16]–[18]. Hence, it might be assumed that this characteristic is also true at the individual level. However, data are essentially lacking on individual telomere attrition rates and its relation to the occurrence of malignancy. Martin-Ruiz et al. did not find an association between telomere length at baseline and malignancy related mortality in a longitudinal study on individuals >85 years old [19].

In the present study, we have investigated individual blood cell telomere shortening in a large cohort of voluntarily donated samples. Our novel results show that the attrition rate was strongly correlated to telomere length at baseline, but unrelated to later tumor development.

## Results

**Figure 1:**
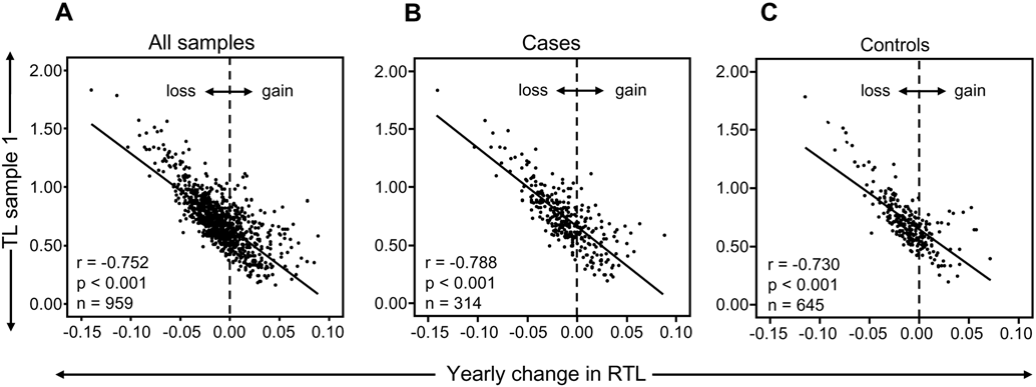
RTL and yearly telomere loss over a 9–11 year period. (A) There was a highly significant and inverse correlation between RTL in sample 1 and telomere attrition rate. The inverse correlation between RTL in sample 1 and attrition rate was similar for (B) future tumor patients and (C) controls.

In the study cohort of 959 individuals, investigated at two occasions with 9–11 year intervals, an overall TL shortening occurred with age as expected (r = −0.164, *P*<0.001) and women displayed longer telomeres than men (*P* = 0.052, after age-adjustments). However, about one third (34%) of all individuals demonstrated a stable TL or even elongated their telomeres over approximately a decade. There were very little differences between cases and controls (31.8% and 34.9%, respectively). When the individual telomere attrition per year was plotted against the relative TL (RTL) value of sample 1, a very strong and inverse correlation was found (r = −0.752, *P*<0.001; n = 959) (Figure 1A). This finding was also observed when analyzing tumor cases (r = −0.788, *P*<0.001; n = 314) and controls (r = −0.730, *P*<0.001; n = 645) separately (Figure 1B and 1C) To make sure that the strong correlation was not based on the very highest or lowest RTL values, the analysis was also made on individuals with RTL values <1 and >0.3. This subcohort showed the same statistical outcome (r = −0.623, p<0.001). Moreover, there was no gender difference (men: r = −0.781, p<0.001, women: r = −0.737, p<0.001). Hence, the attrition rate was most pronounced in individuals displaying the longest telomeres at baseline.

**Figure 2:**
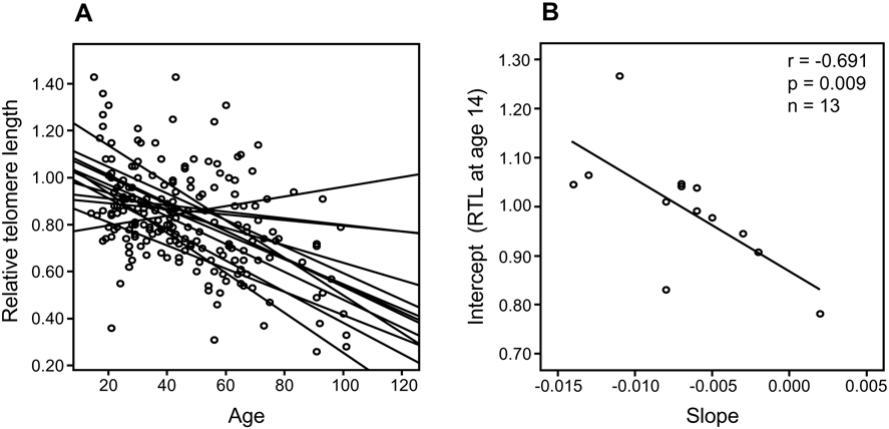
Correlation between RTL and attrition rate in 13 separate families. Each family contained 10 or more (maximum 28) related individuals (i.e., no in-laws). (A) Illustration of telomere shortening with age in 13 individual families. The separate regression lines were obtained by plotting RTL vs. age for each individual in a family. (B) Telomere attrition rate (i.e. slope) in the 13 families in relation to the estimated RTL at age 14 in each family, showing a strong negative correlation.

In a separate cohort of multigenerational families, we selected 13 families encompassing ≥10 members over at least three generations, and plotted RTL against age. A parallellity test revealed statistically significant differences in slope and intercept values between the 13 families (*P*<0.001), i.e. the families differed with regard to telomere shortening over time (Figure 2A). When the slope value from each family was plotted as a function of the corresponding intercept, it was found that RTL at young age (intercept estimated for the age of 14) was highly correlated with the telomere attrition rate (slope) (r = −0.691, *P* = 0.009) (Figure 2B).

**Figure 3:**
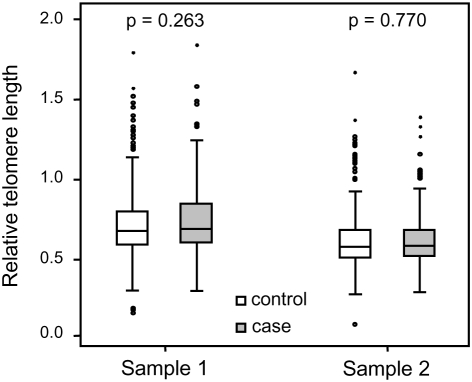
Blood RTL could not predict future tumor development. There was no difference between future tumor patients and controls regarding RTL measured ≥9 years before diagnosis (RTL sample 1) or measured 0–11 years before diagnosis (RTL sample 2).

**Figure 4:**
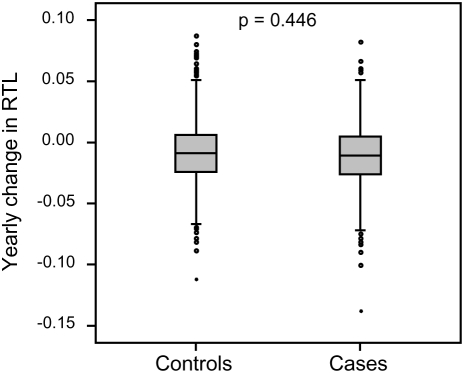
Individual telomere attrition rate was not a marker for future tumor development. Yearly telomere loss at the individual level did not differ between future tumor patients and controls.

**Figure 5:**
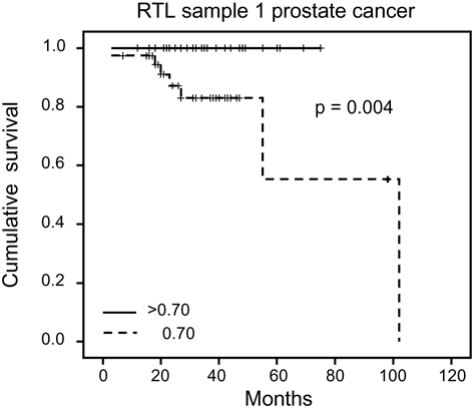
Blood RTL and prognosis in prediagnostic samples from prostate cancer patients. Shorter than median RTL ≥9 years before diagnosis was associated with decreased survival.

RTL in sample 1 or 2 did not differ between cases and controls after age-adjustment (Figure 3), indicating that blood TL was not a potential biomarker for tumor development in prediagnostic samples. The telomere attrition rate was similar for cases and controls (*P* = 0.446 after age and sex adjustments) (Figure 4). As expected, prostate and breast cancer were the most common tumor types (the distribution of different types is given in Table 1), and due to the relatively short follow up time after diagnosis (0–113, mean 34 months) events of deaths were few. When all tumor cases were analyzed as one group, shorter than median RTL (for all tumor cases) in sample 1 (≥9 years before diagnosis) indicated a poor prognosis (not shown in figure). This is illustrated in the largest tumor group, prostate cancer (n = 81), where all deaths were found in the short RTL group in sample 1 (*P* = 0.004; cut off = median RTL value for prostate cancer cases) (Figure 5). When the same analysis was performed for RTL in sample 2 (collected 0–11 years before diagnosis) no significant prognostic difference was found between prostate cancer patients with long versus short telomeres (*P* = 0.174). Moreover, no association between telomere attrition rate and prognosis was found neither in the entire tumor group nor in the prostate cancer group (*P* = 0.266 and *P* = 0.889, respectively).

**Table 1:** Distribution of different tumor types.

Tumor Type	Number	% of Total
Prostate cancer	81	25.8%
Breast cancer	56	17.8%
Colorectal cancer	40	12.7%
Hematopoietic malignancies	33	10.5%
Malignant melanoma	15	4.8%
Endometrial cancer	12	3.8%
Lung cancer	10	3.2%
Liver/Hepatic duct cancer	9	2.9%
Renal cancer	8	2.5%
Brain tumors	7	2.2%
Ovarian cancer	5	1.6%
Others	38	12.1%

## Discussion

Age-associated telomere shortening is a generally accepted finding based on large cross-sectional studies. It has been assumed that this characteristic telomere attrition is true at the individual level as well. It has also been speculated that the telomere attrition rate in blood cells of tumor patients is higher compared to controls. The present longitudinal study cohort demonstrated the expected decline in telomere length by time, but we also observed large individual differences. Actually, in about one third of all individuals an elongation of the telomeres occurred over a decade. Most interestingly, individuals displaying the longest telomeres at the first blood draw demonstrated the most pronounced telomere shortening over time, and vice versa. The average coefficient of variation for the method was ∼6% and some of the variation in TL might be related to the technique itself. However, to make sure that the strong correlation was not based on the very highest or lowest RTL values, the correlation analysis was also made on individuals with RTL values <1 and >0.3. The result of the restricted correlation analysis was very similar to the result of the larger analysis, showing a strong correlation between telomere length at baseline and attrition rate.

A large variation in telomere attrition at the individual level has been observed in previous longitudinal studies on telomere length [19]–[21]. In a very recent study by Aviv et al. [21], TL was measured in leukocytes collected on two occasions from 450 whites and 185 African Americans, participating in the Bogalusa Heart Study. The median time period between the first and second blood sampling was shorter compared to our study (∼6 years vs. ∼10 years), and the participants were fewer and younger (age range: 20.0–40.0 years at baseline). Nevertheless, they found that the age-dependent TL attrition rate was proportional to TL at baseline, which is in accordance to our present study. The majority of participants in their study displayed TL shortening (85.9% of African Americans and 88.0% of whites), whereas the rest displayed a stable or increased TL. Similar to our observation, they also found that the rate of TL shortening varied considerably among individuals.

One explanation to the variations in attrition rate could be differences in epigenetic regulation, with secondary effects on telomere maintenance. Another reason might be that telomerase act preferentially on short telomeres, which has been shown in mice models and cell culture systems [22]–[26]. This would be in line with our observation of a very strong inverse correlation between individual TL at baseline and telomere attrition over time. Interestingly, we found a similar result in our separate family cohort. Hence, comparable data were obtained when analyzing telomere attrition rate at both the individual and at the family level. In the present study, individuals with the shortest TL actually elongated their telomeres over a decade, indicating that the TL maintenance machinery is focused on protecting the shortest telomeres. Nevertheless, other factors are likely to influence the TL attrition rate as well. In our study, the correlation value between blood RTL at baseline (sample 1) and attrition rate was r = −0.752 when analyzing the entire cohort. The corresponding r-squared value is hence 0.566, or ∼57%. This means that the telomere length at time point 1 could explain 57% of the variation in attrition rate. Thus, 43% of the variation might well be explained by other factors, such as life style, oxidative stress, inflammation etc. In the study by Aviv et al. [21], oxidative stress was proposed as a potential candidate for causing proportional telomere shortening. We agree that oxidative stress is likely to be important for telomere attrition, but the theory does not explain why a subset of the cohort demonstrated TL elongation. We suggest that a cellular TL regulating mechanism, rather than environmental/life style factors, is the major factor determining the rate of telomere attrition over time.

The working hypothesis that blood cell TL can indicate a later development of a malignant tumor was not supported in the present study. This hypothesis emanates from data showing altered TL in cases with a variety of malignancies. In urinary bladder, head and neck, lung, and renal cell cancers, shortened blood telomeres have been described at diagnosis, whereas data on breast cancer indicate unchanged or longer telomeres compared to controls [12]–[15]. Since no difference in TL existed between cases and controls, neither ≥9 (sample 1) nor 0–11 (sample 2) years before the appearance of a malignancy, we conclude that blood TL is not a prediagnostic biomarker for malignancy *per se*. However, our cases suffered from a variety of tumors and we cannot exclude that blood TL might be a biomarker for specific tumor types. A support for this is a recent study indicating that short blood telomeres were associated with a decreased risk for melanoma but also an increased risk for basal cell carcinoma, whereas there was no trend for squamous cell carcinoma [27].

In the largest tumor group in our material, prostate cancer, the blood TL ≥9 years before diagnosis seemed to indicate a poor prognosis. All prostate cancer cases with long blood telomeres (>median) were alive five years after diagnosis compared to <60% in the group with short telomeres. However, in the sample taken 0–11 years before prostate cancer diagnosis, TL did not give prognostic information. Thus, since the same prostate cancer patients were studied at different time points ahead of the cancer diagnosis, a shift had occurred indicating differences in the rate of telomere loss. This shift is interesting, since for breast cancer, blood TL at diagnosis seems to be a strong prognostic biomarker, with longer telomeres associated with a worse prognosis [15]. We have obtained similar data for renal cell carcinoma [Svenson et al., unpublished data] indicating that this feature is not unique for breast cancer. Unfortunately, in the present material the other cancer groups were too small to allow this type of statistical calculations. The biological background for these findings is unclear but it is tempting to speculate that factors responding to the presence of a tumor also have an impact on telomere maintenance, especially in immune reactive cells and their precursors. Due to few events in the cancer group a more definitive analysis of prediagnostic TL in relation to prognosis must await longer follow up times.

In conclusion, and similar to what have been observed in cultured cells *in vitro,* human blood cells *in vivo* seem to have a telomere maintenance system that gives priority to short telomeres. Based on human cross-sectional studies of age-associated telomere attrition, it has been speculated that TL at a certain age can predict a theoretical future life span. Our findings indicate that TL regulation through life might be more complex than previously known, complicating such life span predictions. We suggest that, at least in blood cells, the main TL regulator is a general mechanism that senses the telomere length similar to the counting mechanism demonstrated in cells from different species [28]–[31]. However, it might well be possible to avoid excessive telomere loss by living a healthy life as recently indicated [32]. Our data has important implications for our understanding of human telomere biology and for future analyses of telomere maintenance mechanisms *in vivo.*

## Materials and Methods

### Study Cohorts

**Figure 6:**
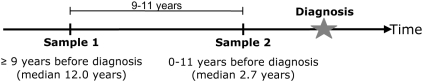
Schematic drawing of blood draws for sample 1 (baseline) and sample 2 (follow up).

The North Sweden Health and Disease Study (NSHDS) include the Västerbotten Intervention Project (VIP), launched in 1985 in the County of Västerbotten, the cardiovascular research program Monitoring of Trends and Determinants in Cardiovascular Diseases (MONICA) and the local Mammography Screening Project (MSP) [33]. At present the population based NSHDS collection contains samples from around 85000 individuals, nearly all Caucasians. Blood was drawn with anticoagulants, separated into plasma, erythrocyte and buffy coat fractions and stored at −80°C in small aliquots. In the NSHDS collection we identified >7000 individuals who had donated blood samples at a ∼10 year interval (9–11 years) and of these 343 persons had obtained a cancer diagnosis after the second blood sample (time from sample 2 to diagnosis: 0–11 years, mean 2.7) (Figure 6). From the same cohort, 686 age and sex matched controls were also selected. The age span was 30–61 years for sample 1 and 40–70 years for sample 2. Cancer cases were identified through record linkages with the regional Cancer Register. Due to insufficient amounts of buffy coat cells for DNA extraction or unsuccessful RT-PCR, 314 cases and 645 controls (totally 1918 samples) were included in the statistical analyses (cases: 176 men and 138 women; controls: 361 men and 284 women).

To permit analysis of a possible family linked pattern regarding TL attrition, a multifamily cohort was also utilized, initially aimed at studying genetic and environmental factors influencing heredity of personality traits, upbringing, general health and longevity (a study designed and conducted in the late 90's by the author RA). In total, whole blood was available from 962 individuals in 68 families (445 men and 517 women) with an age span of 0–102 years. Thirteen of these families could be selected for the purpose of this study (se statistics below).

### Ethics Statement

The study was approved by the Umeå University Ethical Committee.

### Telomere Length Determination

DNA was extracted from buffy coats and whole blood using conventional methods. Relative telomere length was measured using quantitative real-time PCR as described previously [34],[35]. In short, telomeres and a single copy gene (β2-globin) were amplified in all samples including an internal reference control cell line (CCRF-CEM) to which all samples were compared. The ΔΔCt method was used for calculation of RTL values and a standard curve was created in each PCR run to monitor the PCR efficiency. The mean inter-assay coefficient of variation for this method ranges between 4–8% in our laboratory.

### Statistics

Normality was shown regarding RTL distributions. Pearson partial correlation was performed to calculate age-adjusted correlations between continuous variables. ANCOVA was used for age and/or sex adjusted comparisons between groups. Cumulative survival for cancer patients with long vs. short telomeres was investigated using Kaplan-Meier with the log-rank test. Survival was defined as the number of months between diagnosis date to death or to last follow-up (Feb 2008).

To investigate whether the rate of telomere loss with age was linked to TL at a young age, 13 separate families in the multifamily cohort were studied. In each family, samples from 10 or more (maximum 28) related individuals, i.e. no in-laws, were available in at least three generations. The age of the individuals in the youngest generation varied between 14 and 32 years and in the oldest generation between 70 and 101 years. The number of men and women was similar within all families except for one which contained more women. The RTL values were plotted against age and linear regression was used to generate intercept (“starting RTL”) and slope (telomere loss) values for each family. The calculated intercepts corresponded to the estimated RTL value at the age of 14. The slope was then plotted as a function of the intercept and the correlation was examined using Pearson's Correlation Coefficient. MLwiN [36], a software for multilevel analysis, was used to test for parallellity between the 13 regression lines. All other statistics were analyzed in SPSS 15.0. A *P*-value ≤0.05 was considered to be significant.
